# Survival Motor Neuron (SMN) Protein Insufficiency Exacerbates Renal Ischemia/Reperfusion Injury

**DOI:** 10.3389/fphys.2019.00559

**Published:** 2019-05-14

**Authors:** Xiaoqian Qian, Yichao Du, Gengru Jiang, Fujun Lin, Lei Yao

**Affiliations:** ^1^Renal Division, Department of Internal Medicine, Xin Hua Hospital Affiliated to Shanghai Jiao Tong University School of Medicine, Shanghai, China; ^2^Centre for Rare Disease, Shanghai, China; ^3^Sichuan Provincial Academician (Expert) Workstation, The Affiliated Hospital of Southwest Medical University, Luzhou, China

**Keywords:** spinal motor neuron, renal epithelial tubule, ischemia/reperfusion injury, apoptosis, NFκb signaling pathway

## Abstract

The survival of motor neuron (SMN) protein is ubiquitously involved in spliceosome assembly and ribonucleoprotein biogenesis. SMN protein is expressed in kidney and can affect cell death processes. However, the role of SMN in acute kidney injury (AKI) is largely unknown. In the current study, we found that the expression of SMN in the kidney was significantly reduced in both clinical ischemic AKI and a mouse model of renal ischemia-reperfusion injury (IRI). We then used SMN heterozygous knockout (*SMN+/-*) mice and found that the declines in renal function, tubular injury, and tubular cell apoptosis after experimental IRI were significantly more severe in *SMN+/−* mice than those in their wild-type littermates. Concomitantly, the canonical transcription factor nuclear factor-κb (NFκb) signaling was enhanced in ischemic *SMN+/−* mice. *In vitro*, cobalt dichloride (CoCl_2_) treatment reduced SMN expression in proximal tubular epithelial cells. In addition, CoCl_2_-induced apoptosis and activation of NFκb signaling pathway were enhanced by transient transfection of a small-interfering RNA (siRNA) against SMN while attenuated by transient transfection of a full-length SMN plasmid. Taken together, this study for the first time supported the protective role of SMN in ischemic AKI.

## Introduction

Acute kidney injury (AKI) is a common and life-threatening clinical syndrome with adverse outcomes, causing substantial morbidity and mortality in its acute phase (Grams et al., 2011; Lameire et al., 2013). Ischemia-reperfusion injury (IRI) is the major cause of AKI and occurs in many clinical settings, including surgery, organ transplantation, trauma, and shock (Kimura et al., 2011; Xie et al., 2018). IRI involves multiple stresses, including hypoxia, nutrient and growth factor deprivation, energy depletion, oxidant injury, genotoxic stress, endoplasmic reticulum (ER) stress, and other damaging insults, all of which are known to contribute to the induction of renal tubule epithelial cell death, as well as immunological and inflammatory processes (Kaushal and Shah, 2016). It is important to explore the endogenous mechanism of tubular cell injury and death in the pathogenesis of AKI.

The full-length 38 kDa survival of motor neuron (SMN) protein is encoded by the *SMN1* gene, homozygous deletions or mutations of which causes spinal muscular atrophy (SMA), affecting 1 in 6000–10000 live births (Pearn, 1978). The prevalence of SMA carriers in the population is approximately 1/40 (Prior et al., 2010). Although SMN mutations were initially reported to affect the lower motor neurons and muscles in SMA (Crawford and Pardo, 1996), the ubiquitous expression of SMN in the body suggests that SMN may have broad roles in many physiological and pathological processes, as the non-neuronal roles of SMN have been reported in the neuromuscular junction (Kariya et al., 2008; Murray et al., 2008; Kong et al., 2009), muscle (Walker et al., 2008; Martinez-Hernandez et al., 2009; Mutsaers et al., 2011), heart (Bevan et al., 2010; Heier et al., 2010; Shababi et al., 2010), liver (Szunyogova et al., 2016) and pancreas (Bowerman et al., 2014). SMN functions as the modulator of the cell death process and it has been reported that pro-survival pathways are affected by reduced SMN protein levels (Iwahashi et al., 1997; Gangwani et al., 2001; Young et al., 2002; Anderton et al., 2012). A growing body of evidence has highlighted the importance of tubular cell apoptosis as the major pathogenic processes that lead to AKI. However, the anti-apoptosis role of SMN in the kidney remains largely unexplored.

Currently, whether SMN plays a role in ischemia/reperfusion (I/R) -induced kidney injury remains unknown. Therefore, this study was designed to elucidate the role of SMN in ischemia/reperfusion-induced kidney injury. Our results showed that SMN expression was significantly reduced in renal tubular cells of ischemic AKI patients as well as in mice with I/R-induced kidney injury. SMN insufficiency exacerbated I/R-induced kidney injury in heterozygous SMN knockout (*SMN+/−*) mice, along with canonical transcription factor nuclear factor-κb (NFκb) signaling pathway activation, which was verified by *in vitro* experiments, indicating that NFκb signaling pathway may be involved in the process of renal IRI aggravation due to SMN insufficiency.

## Materials and Methods

### Antibodies and Reagents

The primary antibodies used in this study were purchased from the following sources: anti-gemin1 (ab108424, Abcam, United Kingdom), anti-SMN (610646, BD Biosciences, United States), anti-cleaved PARP (ab194217, Abcam, United Kingdom), anti-cleaved caspase-3 (9664, CST, United States), anti-NFκB (8242, CST, United States), anti-phosphor- NFκB (3033, CST, United States), anti-IκBα (4812, CST, United States), anti-phosphor-IκBα (2859, CST, United States), and anti-GAPDH (D110016, Sangon Biotech, China). All secondary antibodies used for immunoblot analysis were from Sangon Biotech. Cobalt dichloride (CoCl_2,_ c8661, United States) was purchased from Sigma-Aldrich.

### Human Studies

#### AKI Patient Samples

AKI was defined on the basis of the Kidney Disease: Improving Global Outcomes (KDIGO) AKI classification and staging system (No Authors, 2012). Paraffin-embedded kidney biopsy samples from 11 patients diagnosed with ischemia AKI and normal kidney sections from 8 normal nephrectomy samples adjacent to tumors used as controls were examined by IHC. These paraffin-embedded kidney biopsies were obtained from a retrospective study investigating biomarkers for AKI and all patients provided written informed consent prior to enrollment. The study protocol was approved by the Institutional Review Board of Xin Hua Hospital Affiliated to Shanghai Jiao Tong University School of Medicine. All study-related procedures were performed in accordance to the Declaration of Helsinki. Patients who developed AKI were classified into three stages. Stage 1 is defined when Scr increases >26 μmol/L within 48 h, or 50–99% from baseline within 7 days, or urine output <0.5 ml/kg per hour for >6 h. Stage 2 is defined when Scr rises 100–199% from baseline within 7 days, or urine output <0.5 ml/kg per hour for >12 h. Stage 3 is defined when Scr rises >200% from baseline within 7 days, or Scr concentration >354 mmol/L with either rise of >26 mmol/L within 48 h or >50% rise from baseline within 7 days, or any requirement for renal replacement therapy, or urine output is, 0.3 ml/kg per hour for 24 h, or anuria for 12 h. Stage 1–2 was defined as moderate group and stage 3 was defined as severe group (Fan et al., 2017).

#### Immunohistochemistry and Analysis

Paraffin-embedded renal biopsy specimens were deparaffinized and incubated with 1 mM EDTA (pH 8.0) at 95–100°C for 1 h for antigen retrieval. After exposure to 3% H_2_O_2_ to block endogenous peroxidase activity and incubation with a buffer containing 5% BSA, 0.1% Triton X-100 and 0.2% milk to reduce non-specific binding, the specimens were incubated with an anti-gemin1 antibody at 4°C overnight and an HRP-conjugated secondary antibody for 1 h at room temperature. Signals of the antigen-antibody complexes were detected with a DAB kit following the protocol of the manufacturer. For quantification, tubular staining was scored separately on a scale of 0–4 (score 0: absence of specific staining; score 1: <25% of the area had specific staining for SMN; score 2: 25–50% of the area had specific staining for SMN; score 3: 50–75% of the area had specific staining for SMN; and score 4: >75% of the area had specific staining for SMN).

### Murine Studies

#### Animals and Ethics

All experiments with mice were approved by the Institutional Animal Care and Use Committee of Xin Hua Hospital, School of Medicine, Shanghai Jiao Tong University. The heterozygous SMN knockout (SMN+/−-) mice (FVB.129P2-Smn1^*tm*1*Msd*^/J, United States) used in this study were obtained from Jackson Laboratories and crossed in our facility. We performed genomic PCR on DNA isolated from the tail (Supplementary Figure S1). Three specific PCR primers were used to detect wild-type (S1, 5′-ATAACACCACCACTCTTACTC-3′, and S2, 5′-GTAGCCGTGATGCCATTGTCA-3′; 1150 bp) and mutant mice (S1 and H1, 5′-AGCCTGAAGAACGAGATCAGC-3′; 950 bp). Adult male wild-type FVB mice were provided by the Laboratory Animal Center of Xin Hua Hospital and were maintained under a 12-h light-dark cycle with free access to food and water.

#### Establishment of an Ischemia/Reperfusion-Induced AKI Model

Eight- to ten-week-old male mice were used for experiments. Renal IRI was induced as described previously (Skrypnyk et al., 2013). Briefly, the mice were anesthetized using pentobarbital sodium (50 mg/kg, i.p.). The right kidney was removed. Blood supply to the left kidney was occluded using a microvascular clamp for 30 min. Body temperature was maintained at 37°C throughout the surgery. Twenty-four hours after reperfusion, the mice were sacrificed. Blood was collected, and isolated serum was stored at −80°C. Kidney tissue samples for histological assessment were fixed in 4% paraformaldehyde (PFA). The remaining kidney tissues were stored at −80°C for gene expression and protein analysis.

#### Renal Function Test

Blood urea nitrogen (BUN) and serum creatinine (Scr) levels were determined using commercial kits from Nanjing Jiancheng Bioengineering Institute (C011-2 and C013-2, Nanjing Jiancheng, China).

#### Hematoxylin-Eosin Staining and Tubular Injury Scoring

The kidneys were fixed with 4% paraformaldehyde and embedded in paraffin. Tissue sections (4 μm) were stained with HE for histological analysis. Tubular injury was scored based on the percentage of tubules that displayed tubular necrosis, cast formation, and tubular dilation (Xu et al., 2015): score 0: normal; score 1: <10%; score 2: 10–25%; score 3: 26–50%; score 4: 51–75%; and score 5: >75%. At least ten randomly selected fields (at 200× magnification) were used per kidney.

#### Immunohistochemical Staining of SMN and Cleaved PARP

The kidney sections were deparaffinized, and after rehydration, antigen retrieval was performed by incubation with 1 mM EDTA (pH 8.0) at 95–100°C for 1 h. Then, the slides were sequentially exposed to 3% H_2_O_2_ to block endogenous peroxidase activity, a buffer containing 5% BSA, 0.1% Triton X-100 and 0.2% milk to reduce non-specific binding, a specific primary antibody at 4°C overnight, and an HRP-conjugated secondary antibody for 1 h at room temperature. Signals of the antigen-antibody complexes were detected with a DAB kit following the protocol of the manufacturer. Finally, the slides were counterstained with hematoxylin.

#### TUNEL Staining

Cell death in the kidney was detected by Transferase-mediated deoxyuridine triphosphate-biotin nick end labeling (TUNEL) staining (12156792910, Roche Life Science, Germany), as described previously (Livingston et al., 2016). Positive staining with nuclear DNA fragmentation was detected by fluorescence microscopy and quantified by the number of TUNEL-positive cells in ten fields per section, with at least three sections per kidney.

#### Cell Culture

Mouse renal proximal tubule epithelial cells (mTECs) were obtained from the Cell Bank of the Chinese Academy of Sciences (Shanghai, China). mTECs were maintained in DMEM (Sigma, United States) supplemented with 10% fetal bovine serum (Gibco, United States), penicillin (100 U/ml) and streptomycin (100 μg/ml) at 37°C with 5% CO_2_ in a humidified incubator.

#### Cell Transfection and Treatment

Cells were treated with cobalt dichloride (CoCl_2_) to induce cellular injury. Mouse FL-SMN cDNA fragments were cloned into the pcDNA3 vector, which was purchased from Servicebio (Wuhan, China). Cells were seeded into 6-well plates at 80% confluency and transfected with the indicated plasmids using Lipofectamine 2000 (Invitrogen, United States) for 72 h. An SMN small interfering RNA (siRNA) and a control RNA with a scrambled sequence were synthesized by RiboBio (Guangzhou, China). mTECs were transfected with the siRNAs using RFect Transfection Reagent (11013, BAIDAI, China) for 72 h. Prior to cell collection, some cells were treated with CoCl_2_ (300 μM) for 24 h. The cells were then lysed and processed for protein analysis or apoptosis assays.

#### Annexin V/PI Double Staining

The cells were trypsinized and washed three times with PBS, centrifuged (2000 rpm at room temperature) for 2 min, adjusted to 5 × 10^4^/ml and double-stained with annexin V-FITC and PI (Annexin V-FITC Apoptosis Detection Kit, BD Biosciences, United States). After incubation for 15 min at room temperature in the dark, the fluorescence intensity was measured using CytoFLEX (Beckman, United States).

#### Real-Time PCR

Total RNA was isolated from cells or kidneys using TRIzol Reagent (Takara, Japan) according to the manufacturer’s instructions. cDNA was synthesized using PrimeScript RT Master Mix (RR036, Takara, Japan). Real-time PCR was performed using SYBR Premix Ex Taq (RR420, Takara, Japan) and probed on an ABI Prism 7500 Sequence Detection System. The mRNA levels were normalized to 18S rRNA. The forward primer of KIM-1 is GTAGCTGTGGGCCTTGTAGTT and the reverse primer is CCTATCAGAAGAGCAGTCGGTA.

#### Western Blot Analysis

Frozen kidney tissues or cultured mTECs were lysed with RIPA lysis buffer (Beyotime, China) containing 1 mM PMSF (Beyotime, China). Equal amounts of protein were separated by 8% or 12% SDS-PAGE and transferred onto PVDF membranes. The membranes were blocked with 5% fat-free milk and subsequently probed with primary antibodies overnight at 4°C, followed by anti-mouse or anti-rabbit horseradish peroxidase-conjugated secondary antibodies (1:1000), and developed by ECL (EMD Millipore, United States). Densitometry analysis was performed using ImageJ software.

#### Statistical Analysis

Data are presented as the mean ± SD from at least triplicate experiments unless otherwise indicated. The statistical significance of differences was examined by one-way analysis of variance (ANOVA) followed by Student’s *t*-test using GraphPad Prism 7 software. *p* < 0.05 was considered statistically significant.

## Results

### Reduction of SMN in the Renal Tubular Cells in Ischemic AKI Patients and Mice With IRI

**FIGURE 1:**
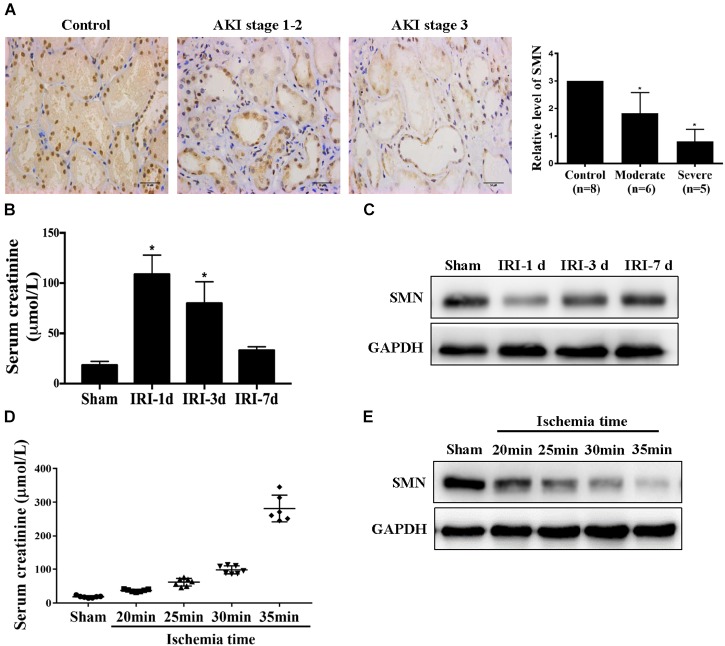
SMN expression in the kidney in patients and IRI mice. **(A)** Immunostaining of SMN in normal kidney and ischemic AKI kidney sections. Representative images of nephrectomy samples from one patient in each of three groups: stage 1–2 (*n* = 6), stage 3 (*n* = 5), and normal kidney (*n* = 4). Original magnification, 400×. Bar = 50 μm. Semiquantitative analysis of tubular SMN staining. Data are expressed as the mean ± SD. (^∗^*p* < 0.01 vs. normal samples). WT mice were subjected to uninephrectomy and I/R as described in Methods. The serum and remaining kidney were collected on day 1, day 3 or day 7 after I/R. **(B)** Serum creatinine (Scr); **(C)** SMN protein levels were determined by Western blot assay (^∗^*p* < 0.05 vs. sham-operated WT mice, *n* = 5). **(D)** Serum creatinine (Scr), and **(E)** Western blot analysis of SMN protein expression in I/R-induced AKI in wild-type mice for different ischemia time. GAPDH was used as loading control. Data are expressed as the mean ± SD. (*^∗^p* < 0.05 vs. sham-operated WT mice, n ≥ 6).

To investigate the possible role of SMN in ischemic AKI, we first determined tubular SMN expression by immunohistochemistry on biopsy materials from patients diagnosed with ischemia-induced AKI (*n* = 11). Nephrectomy specimens from patients with normal kidney function were used as controls (*n* = 8). Figure 1A shows significantly reduced SMN staining in tubular epithelial cells from AKI patients compared to those in controls (control vs. moderate, *p* = 0.016; control vs. severe, *p* = 0.0002). Additionally, SMN expression was significantly reduced in patients with stage 3 AKI than those in patients with stage 1–2 AKI (moderate vs. severe, *p* = 0.023).

Then, we examined SMN expression in kidneys in male wild-type (WT) FVB mice suffering I/R-induced injury. Scr confirmed I/R-induced renal function damage in the mice, as shown in Figure 1B. Scr reached the highest level at day 1 post IRI, and gradually declined from day 3 to day 7 post IRI. Western blot showed that the SMN protein level in the I/R group was significantly reduced at day 1 after IRI, compared with that in the sham group and recovered at 3 and 7 days post IRI (Figure 1C). Next, we examined the SMN expression in the kidneys for different ischemia time at 1 day after IRI. Scr levels were upregulated in a time-dependent manner (Figure 1D). Along with Scr elevation, SMN expression was decreased (Figure 1E).

### SMN Insufficiency Exacerbated Renal Injury After Ischemia/Reperfusion (I/R)

**FIGURE 2:**
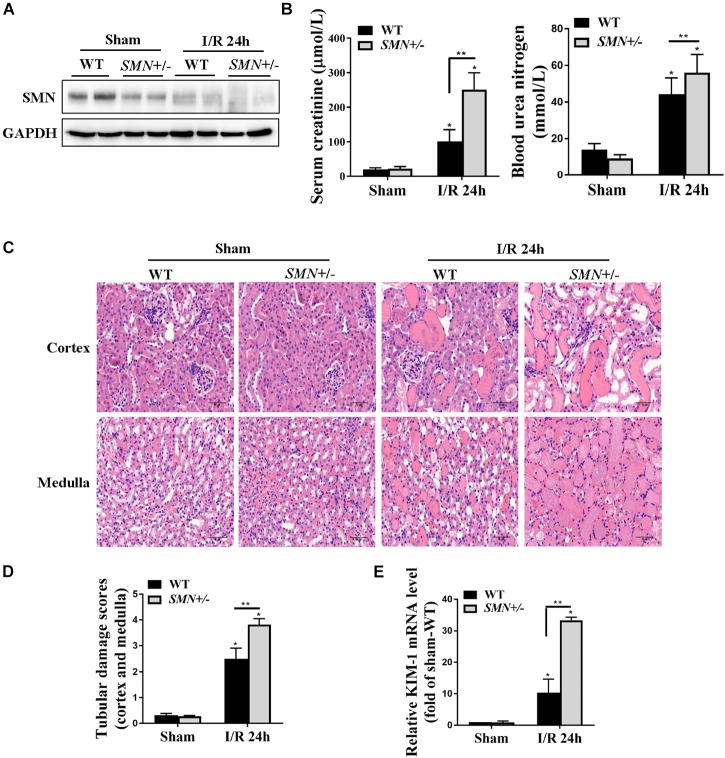
Assessment of kidney function and renal histological injury in WT and SMN+/– mice subjected to IRI. The I/R-induced AKI mouse model was induced in WT and SMN+/– mice. Blood and kidney tissues were collected at 24 h after I/R, as described in the Materials and Methods. **(A)** Western blot analysis of SMN protein expression in I/R-induced AKI in WT and *SMN+/–* mice. GAPDH was used as the loading control. **(B)** Serum creatinine (Scr, *n* = 10) and blood urea nitrogen (BUN, *n* = 8). **(C)** Representative images of hematoxylin and eosin (HE) staining. Original magnification, 400×. Bar = 50 μm. **(D)** Semiquantitative assessment of tubular damage. WT sham, *SMN+/–* sham, and *SMN+/–* IRI, *n* = 8; WT IRI, *n* = 11. **(E)** Real-time PCR analysis of kidney KIM-1 mRNA expression. Data are expressed as the mean ± SD; (^∗^*p* < 0.0001 vs. sham-operated WT mice; ^∗∗^*p* < 0.05 vs. ischemic WT mice under the same experimental conditions).

We next examined I/R-induced AKI in heterozygous SMN knockout (*SMN+/−*) mice. At the age of eight- to ten-week-old, male WT and *SMN+/−* mice were subjected to ischemia for 30 min, and the kidneys and serum were collected at 24 h after reperfusion. Western blotting showed that, compared to that in WT mice, SMN protein abundance in the kidneys of *SMN+/−* mice were significantly reduced prior to ischemia. The protein levels of SMN in the kidneys of *SMN+/−* mice post I/R were also remarkably reduced compared to those in their wild-type littermates (Figure 2A). Serum Cr levels were comparable prior to I/R but significantly higher in *SMN+/−* mice at 24 h after reperfusion (101.3 ± 34 vs. 250.8 ± 49 μmol/L in the wild-type control; *n* = 10, *p* < 0.0001; Figure 2B). BUN was 56 ± 9.9 mmol/L in *SMN+/−* mice, which was 26.5% higher than that in their wild-type littermates (*n* = 8, *p* = 0.011; Figure 2B). In *SMN+/−* mice subjected to the sham surgery, the kidneys displayed apparently normal morphology and function. In comparison to that in the wild-type control, the extent of IRI injury was more severe in *SMN+/−* mice, as indicated by tubule dilatation, tubule detachment, cast formation, and the presence of inflammatory cells (*n* = 8 or 11, *p* < 0.0001; Figure 2C,D). Kidney injury molecule 1 (KIM-1) is a transmembrane tubular protein and increased KIM-1 expression is correlated with the degree of renal damage (Schrezenmeier et al., 2017). We assessed KIM-1 mRNA expression levels by real-time PCR. Figure 2E demonstrates that KIM-1 mRNA was more abundant in the kidney in the *SMN+/−* mice subjected to IRI than that in the WT mice (*n* = 6 or 8, *p* < 0.0001).

### Increased I/R-Induced Renal Apoptosis in *SMN+/−* Mice

**FIGURE 3:**
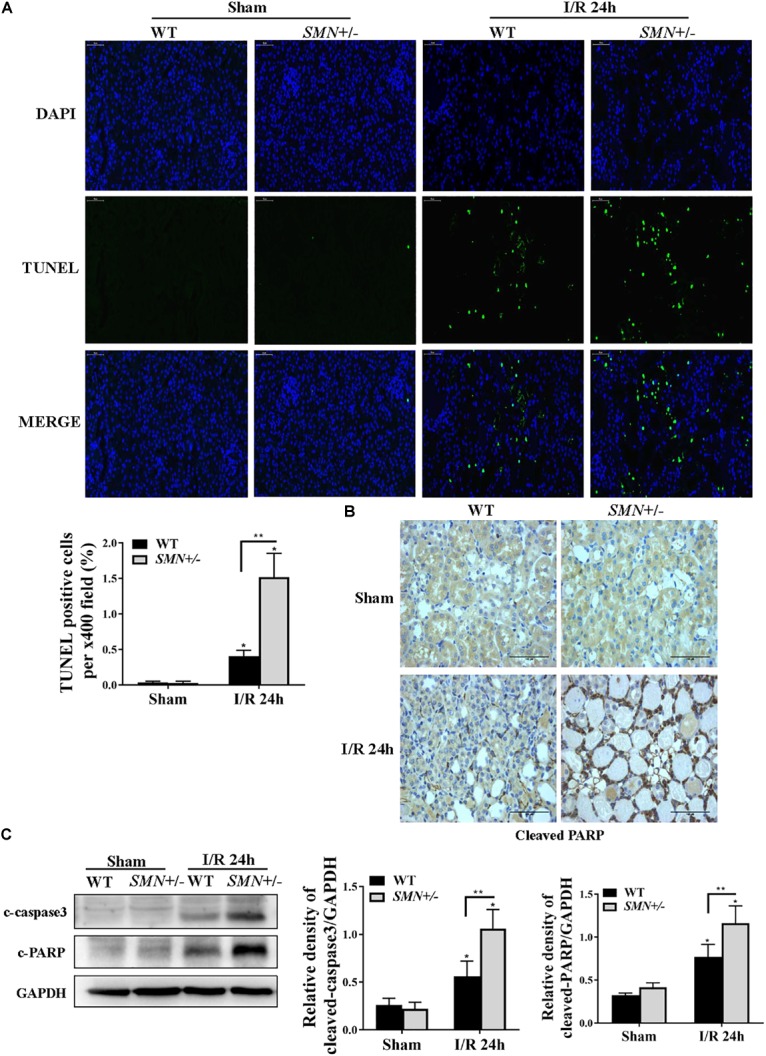
Comparison of renal tubular apoptosis between WT and *SMN+/–* mice subjected to IRI. The I/R-induced AKI mouse model was generated in WT and *SMN+/–* mice. Kidney tissues were collected 24 h after I/R. **(A)** Representative images of TUNEL staining and overlays with DAPI staining. Original magnification, 400×. Bar = 50 μm. Quantitative analysis of TUNEL-positive cells; (^∗^*p* < 0.0001 vs. sham-operated WT mice; ^∗∗^*p* < 0.0001 vs. ischemic WT mice under the same experimental conditions; *n* = 4). **(B)** Representative images of immunohistochemical staining with an anti-cleaved PARP antibody. Original magnification, 400×. Bar = 50 μm. **(C)** Western blot analysis of apoptotic protein markers. Kidney tissue lysates were probed with anti-cleaved caspase-3 (c-caspase-3) and cleaved PARP (c-PARP) antibodies. Relative protein abundance was semi-quantified by densitometry (*n* = 7 or 3). Data were expressed as the mean ± SD (^∗^*p* < 0.05 vs. sham-operated WT mice, ^∗∗^*p* < 0.05 vs. ischemic WT mice under the same experimental conditions).

Cell death is one of the important characteristics of I/R induced AKI. We first defined the effects of SMN-mediated cell apoptosis in the setting of I/R-induced AKI by TUNEL staining. As demonstrated in Figure 3A, compared to that in WT mice, the number of TUNEL-positive cells in the *SMN+/−* group mice after IRI were significantly increased (*p* < 0.0001). Immunohistochemistry analysis of kidney tissues with an anti-cleaved poly ADP-ribose polymerase (PARP) antibody showed an increase in cleaved PARP staining in tubular cells in IR-induced AKI in *SMN+/−* mice compared to those in WT I/R mice (Figure 3B). This finding was further confirmed by Western blot analysis with increased cleaved caspase-3 and cleaved PARP protein expression 24 h after I/R-induced injury in kidney tissues of *SMN+/−* mice compared to those in WT I/R mice (Figure 3C).

### SMN Insufficiency Lead to Aggravated Cytotoxicity and Activation of NFκb Signaling Pathway Upon Cobalt Chloride Treatment in Cultured mTECs

**FIGURE 4:**
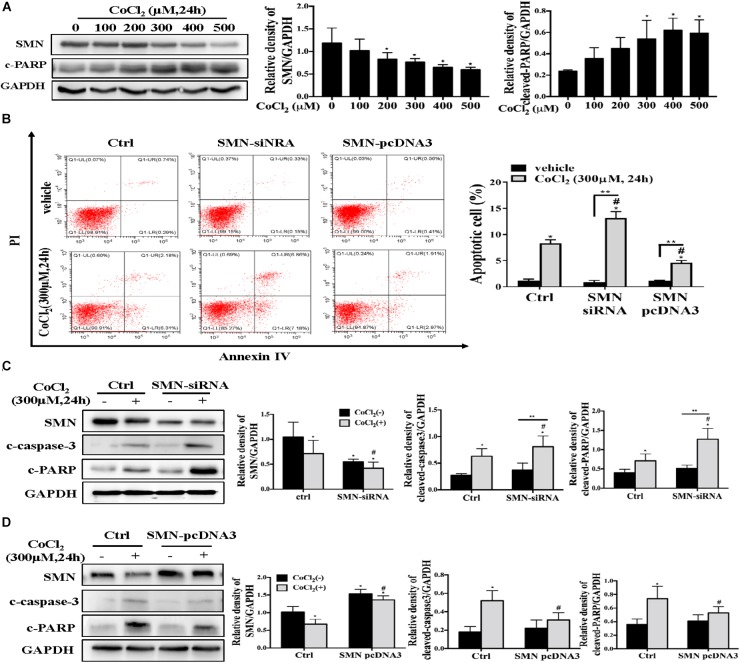
SMN expression affects hypoxia-induced apoptosis in mTECs. **(A)** mTECs were treated with different concentrations of CoCl_2_ for 24 h. SMN and cleaved PARP protein levels were assessed by Western blotting. **(B)** mTECs were transfected with an SMN small interfering RNA or the SMN-pcDNA3 plasmid. After 72 h, the cells were treated with or without CoCl_2_ (300 μM) for 24 h. Apoptotic cells were detected by flow cytometric analysis of PI and annexin V double staining. **(C)** mTECs were transfected with a control (ctrl) or SMN siRNA. After 72 h, the cells were treated with or without CoCl_2_ (300 μM) for 24 h. The protein levels of SMN, cleaved PARP, and cleaved caspase-3 were determined by Western blot analysis. **(D)** Cells were transfected with control (ctrl) or pcDNA3-SMN. After 72 h, the cells were treated with or without CoCl_2_ (300 μM) for 24 h. The protein levels of SMN, cleaved PARP, and cleaved caspase-3 were determined by Western blot analysis. Representative data from at least 3 independent experiments are shown. Data are expressed as the mean ± SD (^∗^*p* < 0.01 vs. control without CoCl_2_ treatment; ^#^*p* < 0.05 between groups under the same experimental conditions; ^∗∗^*p* < 0.05).

To mimic hypoxia *in vitro*, mouse proximal tubule epithelial cells (mTECs) were exposed to hypoxia-inducible factor 1 (HIF1) α-stabilizing agent cobalt chloride (CoCl_2_). Exposure of mTECs to CoCl_2_ treatment for 24 h at concentrations from 100 to 500 μmol/L substantially reduced the protein expression of SMN in a dose-dependent manner (Figure 4A). Along with the reduced expression of SMN, cleaved PARP protein levels were upregulated in a dose-dependent manner (Figure 4A). Then, we manipulated SMN protein expression in mTECs by either knocking down endogenous SMN through siRNA or overexpressing SMN by transfecting the SMN plasmid. 300 μmol/L CoCl_2_ significantly increased the number of annexin V-positive cells, and this effect was further exacerbated by the transfection with SMN-siRNA, whereas overexpressing SMN significantly decreased the number of annexin V-positive cells caused by CoCl_2_ treatment (Figure 4B). Western blot analysis also demonstrated the aggravated CoCl_2_-induced apoptosis when endogenous SMN was knocked down, as evidenced by increased cleaved caspase-3 and cleaved PARP expression in SMN-siRNA-transfected cells (Figure 4C). In contrast, SMN overexpression reduced the expression of cleaved caspase 3 and cleaved PARP in mTECs treated with CoCl_2_ (Figure 4D).

**FIGURE 5:**
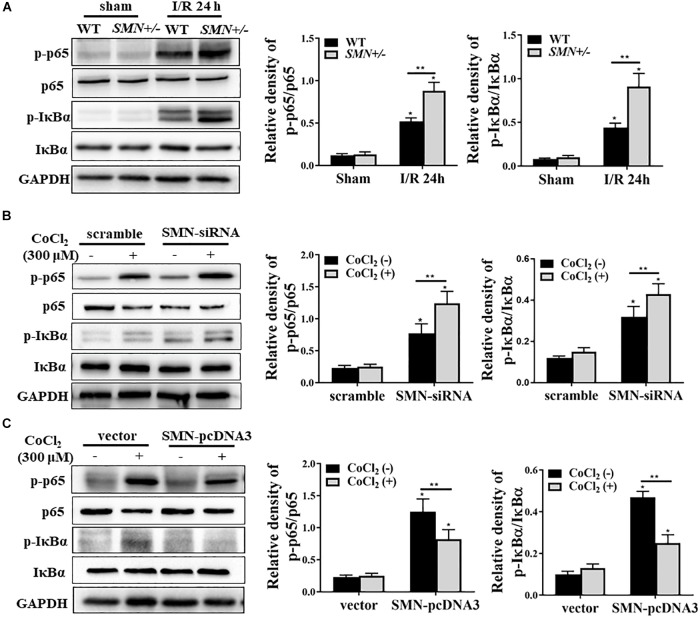
SMN regulates NF-κB signaling pathway in ischemia AKI. **(A)** Representative western blot and summarized data showing the protein levels of NF-κB (p65), IκBα, p-NF-κB (p-65), and p-IκBα in the kidney from WT and *SMN*+/– mice after 24 h of I/R injury. **(B)** mTECs were transfected with control (ctrl) or SMN-siRNA. After 72 h, the cells were treated with or without CoCl_2_ (300 μM) for 24 h. The protein levels of NF-κB (p65), IκBα, p-NF-κB (p-65), and p-IκBα were determined by Western blot analysis. **(C)** Cells were transfected with control (ctrl) or pcDNA3-SMN. After 72 h, the cells were treated with or without CoCl_2_ (300 μM) for 24 h. The protein levels of NF-κB (p65), IκBα, p-NF-κB (p-65), and p- IκBα were determined by Western blot analysis. Representative data are shown from at least 3 independent experiments. Quantification is shown as the mean ± SD; (^∗^*p* < 0.05 vs. control ^∗∗^*p* < 0.05 between groups under the same experimental conditions).

NF-κB is a ubiquitously expressed transcription factor system and as a key regulator in many cellular processes, including inflammatory response and apoptosis (Marko et al., 2016). We examined the total and phosphorylated NFκb and IκBα augmentation under SMN insufficiency conditions post I/R injury. Consistent with previous studies, I/R injury induced NFκb activation in kidney tissues of both WT and *SMN+/−* mice, but more remarkable in those of *SMN+/−* mice (Figure 5A). *In vitro* experiments consistently showed more prominant upregulation of phosphorylated NFκb and IκBα expression in cultured mTECs subjected to siRNA-SMN treatment under CoCl_2_ treatment (Figure 5B). In contrast, overexpression of SMN inhibited CoCl_2_-stimulated activation of NFκb signaling pathway (Figure 5C).

## Discussion

In this work, we identify a novel role of SMN in modulating the development of ischemic AKI. The major findings included: (1) SMN levels were significantly reduced in renal tubular cells in biopsy samples from patients diagnosed with ischemic AKI and in renal tubular cells from an IRI mouse model. (2) The degree of reduced SMN expression was correlated with the degree of renal function impairment. (3) SMN insufficiency exacerbated ischemic AKI as *SMN+/−* mice displayed more remarkable I/R-induced tubular apoptosis and inflammatory response along with NFκb signaling activation, compared to the WT control mice post IRI. Meanwhile, CoCl_2_-induced cytotoxicity and activation of NFκb signaling pathway in mTECs *in vitro* were attenuated after overexpressed with SMN. Taken together, these results suggested that SMN possesses an important renoprotective role from ischemic renal injury by modulating apoptosis, which play a key role in the pathophysiology of I/R injury.

A growing body of evidence has highlighted the importance of tubular cell injury and apoptosis as the major pathogenic processes that lead to AKI. As an important mediator of cell fate, the ability of the SMN protein to regulate cell survival and apoptosis was supported by several studies, including reports of defective cell apoptosis in cultured SMN-depleted cells (Ilangovan et al., 2003) and SMN deficiency-activated apoptosis-like cell death in neuronal cells (Gallotta et al., 2016). In addition to the involvement of SMN in the neuromuscular system, low levels of SMN have been reported to promote cardiomyocyte apoptosis (Sheng et al., 2018). To date, the specific anti-apoptotic mechanism of SMN remains unknown, although it is generally accepted that the anti-apoptosis effect of SMN is mainly mediated by blocking caspase-3 activation and regulating other key regulators of cell survival, such as Bcl-2, p53, and Bcl-xL (Iwahashi et al., 1997; Gangwani et al., 2001; Young et al., 2002; Anderton et al., 2012). In this study, we found that SMN insufficiency leads to the activation of the caspase-3 subunit both *in vivo* and *in vitro*. SMN deficiency also results in poly (ADP-ribose) polymerase (PARP) cleavage in response to hypoxia. PARP is a key mediator of cell death in the DNA damage response (DDR). Interestingly, severe DNA damage can induce both the extrinsic and intrinsic apoptosis pathways. During this process, caspase-3 plays a central role in the execution of the apoptotic program and is responsible for the cleavage of PARP. Thus, it is possible that the effect of SMN on cell apoptosis may be involved in the DDR process during AKI. Nevertheless, as the terminal stage of apoptosis, caspase-3 activation mediates both the intrinsic or extrinsic apoptosis pathway. The extrinsic pathway is activated by cell surface death receptors, and the intrinsic apoptosis pathway is activated by mitochondrial damage (Havasi and Borkan, 2011). Therefore, the mechanisms underlying the effect of SMN on the extrinsic or intrinsic apoptosis pathway in AKI should be further explored.

NF-κB is a ubiquitously expressed transcription factor system and as a key regulator of innate and adaptive immunity, which turns on a variety of genes to participate in many cellular processes, including inflammatory response, apoptosis, and cell proliferation and differentiation (Marko et al., 2016). The NF-κB family consists of homodimers and heterodimers of five members: RelA (p65), RelB, c-Rel, NF-κB1 (p50), and NF-κB2 (p52) (Ghosh and Hayden, 2012). The NF-κB pathway can be activated by canonical or non-canonical route. A growing body of evidence highlights the importance of canonical NF-κB in the pathogenesis of AKI. Lajos et al. demonstrated that activating NF-κB signaling in renal tubular cell exacerbated tubular injury, apoptosis and necrosis after ischemic AKI induction (Marko et al., 2016). Ozkok et al. (2016) also reported that NF-κB transcriptional inhibition ameliorated kidney function and tubule epithelial cells injuries and cell death in cisplatin-induced AKI. Consistent with these previous studies, we observed the activation of NF-κB signaling in renal tubular cells after ischemic AKI induction. Notably, we found that SMN insufficiency enhanced NF-κB activation both *in vivo* and vitro, as evidenced by the upregulation of p-NF-κB (p-65) and p-IκBα protein levels. In contrast, SMN overexpression attenuated CoCl_2_-induced activation of NFκb signaling pathway. In motor neurons, SMN prevented NF-κB activation by blocking TRAF6-mediated IKK polyubiquitination and thereby inhibited p65-mediated canonical NF-κB pathway activation (Kim and Choi, 2017). In AKI, whether SMN is a key mediator of NF-κB activation or the effect of SMN on NFkB is secondary to its effect on tubular damage remains to be investigated in further studies.

SMN+/− carriers, with the prevalence of 1/40 in the population (Prior et al., 2010) are clinically unaffected throughout their lives. In this study, *SMN*+/− mice with SMN haploinsufficiency, also displayed a life span similar to that of their wild-type littermates, and they were also phenotypically normal and had no appreciable defects in kidney morphology or function. Notably, *SMN*+/− mice had exacerbated tubular injury changes post IRI, indicating that *SMN*+/− carriers might be susceptible to ischemic AKI, which needs to be confirmed in further prospective studies. In addition, although we demonstrated *in vitro* that SMN-knockdown cultured mTECs treated with hypoxia-mimetic agent CoCl_2_ lead to aggravated cytotoxicity and activation of NFκb signaling pathway, CoCl_2_ might only mimic part of the hypoxia response and it cannot be rule out that CoCl_2_ exerted a direct toxic effect that lead to cell death (Munoz-Sanchez and Chanez-Cardenas, 2019). Hypoxia chamber experiment needs to be carried out further to solid the results found in this study.

## Conclusion

In conclusion, this study uncovered the underlying protective role of SMN against ischemia-induced tubular injury and renal tubule epithelial cell apoptosis, through mechanisms involving NFκb signaling pathway.

## Data Availability

All datasets generated for this study are included in the manuscript and/or the Supplementary Files.

## Ethics Statement

This study was carried out in accordance with the recommendations of the Institutional Animal Care and Use Committee of Xin Hua Hospital, School of Medicine, Shanghai Jiao Tong University. The protocol was approved by the Institutional Animal Care and Use Committee of Xin Hua Hospital, School of Medicine, Shanghai Jiao Tong University. The study protocol was approved by the Institutional Review Board of Xin Hua Hospital Affiliated to Shanghai Jiao Tong University School of Medicine. All study-related procedures were performed in accordance to the Declaration of Helsinki.

## Author Contributions

GJ, FL, and LY designed the study and interpreted the results and revised the manuscript. XQ conducted most of the experiments and drafted the manuscript. YD performed parts of cellular experiments. XQ and FL were analyzed the data.

## Conflict of Interest Statement

The authors declare that the research was conducted in the absence of any commercial or financial relationships that could be construed as a potential conflict of interest.
