# Supplementary material for: Survival Motor Neuron (SMN) Protein Insufficiency Exacerbates Renal Ischemia/Reperfusion Injury
Source: Front Physiol. 2019 May 14;10:559. doi: 10.3389/fphys.2019.00559 (PMC6527877; doi:10.3389/fphys.2019.00559)
Supplement: Supplementary file 1 [file Image_1.pdf]

Supplementary Figure

Supplementary Figure 1.

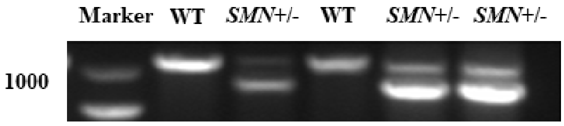

Supplementary Figure 1. Genotyping of *SMN* mice. PCR genotyping of the offspring from *SMN*+/- x *SMN*+/+ with primers S1, S2 and H1.
